# One-reactor plasma assisted fabrication of ZnO@TiO_**2**_ multishell nanotubes: assessing the impact of a full coverage on the photovoltaic performance

**DOI:** 10.1038/s41598-017-09601-7

**Published:** 2017-08-29

**Authors:** Alejandro Nicolas Filippin, Manuel Macias-Montero, Zineb Saghi, Jesús Idígoras, Pierre Burdet, Juan R. Sanchez-Valencia, Angel Barranco, Paul A. Migdley, Juan A. Anta, Ana Borras

**Affiliations:** 1 Nanotechnology on Surfaces Laboratory, ICMS Materials Science Institute of Seville (CSIC-US). C/Americo Vespucio 49, 41092 Seville, Spain; 2 Department of Materials Science and Metallurgy, University of Cambridge, 27 Charles Babbage Road, CB3 0FS, Cambridge, United Kingdom; 3 Departamento de Sistemas Físicos, Químicos y Naturales, Universidad Pablo de Olavide, Carretera de Utrera km 1, 41013 Seville, Spain; 4 University of Grenoble Alpes, Grenoble F-38000, France; CEA, LETI, MINATEC Campus, Grenoble, F- 38054 France

## Abstract

This paper addresses the fabrication of vertically aligned ZnO@TiO_2_ multishell nanotubes by a combined full vacuum-plasma approach at mild temperatures. The growth is carried out within the premises of a one-reactor approach, i.e. minimizing the number of vacuum chambers and sample transferences. In this way, the interface between ZnO and TiO_2_ is fully preserved from humidity thus increasing ZnO durability and stability. These nanostructures are studied by scanning electron microscopy (SEM), scanning transmission electron microscopy (STEM) and energy dispersive X-ray spectroscopy in STEM (EDX-STEM). High density one-dimensional arrays of these nanotubes formed on FTO substrates are applied as photoanode in a dye-sensitized solar cell (DSC). The evolution of the dye adsorption capacity and solar cells parameters are explored as a function of the crystallinity and thickness of the TiO_2_ shell. The results show the critical effect of a full coverage by TiO_2_ of ZnO core to explain the mixed results found in the literature.

## Introduction

In a recent work, we addressed the fabrication of ZnO nanotubes by an all-vacuum template procedure^1^. The protocol involves 3 basic steps: (i) growth of single crystal organic nanowires (ONWs) by Physical Vapor Deposition (OPVD)^2^ acting as supported 1D templates; (ii) formation of a metal oxide shell by Plasma Enhanced Chemical Vapor Deposition (PECVD)^3, 4^ and, (iii) sublimation of the organic template to yield metal oxide nanotubes. We aim herein to extend the potential of the developed protocol for the fabrication of multishell ZnO@TiO_2_ nanotubes. Critical advantages of this methodology are its general character from the point of view of both substrates and shells materials and nanostructure, mild experimental conditions for organic template and shell deposition as well as template removal. In addition, this protocol allows the application of a one-reactor approach, i.e. minimizing the number of vacuum/plasma chambers and transferences and thus avoiding exposure of the different layers/shells to air. We borrow this name from synthetic chemistry where there is a trend towards coupling several steps of a multi-step chemical reaction into just one reactor. This one-pot strategy is of high interest for the chemical industry due to economic and environmental advantages^5^. In this way, a methodology integrating sequential and/or simultaneous fabrication/processing steps without the necessity of exposing clean surfaces to ambient conditions and minimizing transfer between chambers can be regarded as one-reactor strategy. This approach provides an unparalleled accurate control on the interfacial composition of sequentially deposited materials including sharp interfaces between two photofunctional metal oxides such as ZnO and TiO_2_, preserving the surfaces and interfaces and reducing the use of solvents. In order to show these promising advantages, we will thoroughly characterize the ZnO@TiO_2_ NTs system by advanced scanning and transmission electron microscopies allowing a unique and deeper insight into the distribution of materials at the nanoscale, the state and sharpness of the interfaces and, degree of conformality of the shell(s)^1, 6, 7^.

In the second part of the article, we will demonstrate the stability of the NTs under room conditions and their straightforward implementation into photoelectric devices showing the fabrication of ZnO@TiO_2_ dye-sensitized solar cells.

Dye-sensitized solar cells (DSCs) efficiency has slowly improved from 10% to 13% in a time lapse of 20 years^8, 9^. New ruthenium-free dyes and combination of dyes have been introduced in order to enhance light harvesting (boosting the photocurrent) while in conjunction with novel Co(II/III) electrolytes a parallel increase of the photovoltage has been favored^10^. Furthermore, a great deal of research has been carried out to optimize the photoanode of DSCs through the choice of the active material and its nanostructuration. In this regard, two main active photoanode materials have been widely studied for this application: ZnO and TiO_2_^5, 11–17^. Bulk wurtzite ZnO has a direct band gap of 3.44 eV and n-type behavior. Even if the causes for such a n-type behavior are still unclear, it is speculated that the unintended incorporation of impurities such as H would be a possible explanation^18^. The high bulk electron mobility of ZnO, μ_e_ = 200 cm^2^V^−1^s^−1^, and acceptable exciton binding energy of 60 meV make it an attractive candidate for its potential implementation in DSCs^13, 14, 19, 20^. However, its known degradation by many usual dyes, its instability in aqueous solution and the poor charge-separation potential (injection) at the dye/oxide interface, limit the overall cell performance and operation lifetime^21^. On the other hand, TiO_2_ was the first choice for DSCs since it is chemically stable and it possesses an injection efficiency of 100%, twice that of ZnO. Moreover, bulk TiO_2_ has a band gap of 3.2 eV for the anatase phase and like ZnO it is also an n-type semiconductor due to oxygen vacancies and the presence of Ti^3+^ cations^8, 22^. The major drawbacks associated with TiO_2_ are its low electron mobility, ranging from 1 cm^2^V^−1^s^−1^ for amorphous TiO_2_ to 30 cm^2^V^−1^s^−1^ for anatase, and its relatively low exciton binding energy of 4 meV^23, 24^.

The superior performance of these 1D ZnO nanostructures in DSCs when compared to mesoporous thin films of the same material was demonstrated by Law *et al*.^25^ and adjudged to the intrinsic higher specific area of the 1D material. Although ZnO NTs effectively outperformed their thin film counterpart, the already mentioned poor chemical stability of ZnO certainly limits its use in DSCs regardless of the microstructure/nanostructure. In an attempt to hinder its degradation, several researchers have placed their efforts in the synthesis of ZnO@TiO_2_ core@shell nanostructures for DSCs, generally obtaining mixed results^26–31^. In fact, Yang *et al*. unraveled the unexpected long-term instability of ZnO nanowires protected by a TiO_2_ shell formed by atomic layer deposition (ALD)^32^. They showed an enhanced photoetching and self-induced photocorrosion of c-(0001) oriented ZnO/TiO_2_ NWs stored under ambient conditions and exposed to UV-light. In our case, the polycrystalline character of the ZnO shell will play an advantageous role in the stability of the ZnO-TiO_2_ interface. Thus, we have prepared 1D-based DSCs comprising ZnO NTs covered with a TiO_2_ shell following the same multi-step produce described above and investigated the effect of this outer shell crystallinity and thickness on the cell performance. Moreover, a potential explanation to the observed discrepancies in the literature concerning ZnO@TiO_2_-based DSCs is given.

## Results and Discussion

### Advanced Characterization of the Multishell Nanotubes

**Figure 1:**
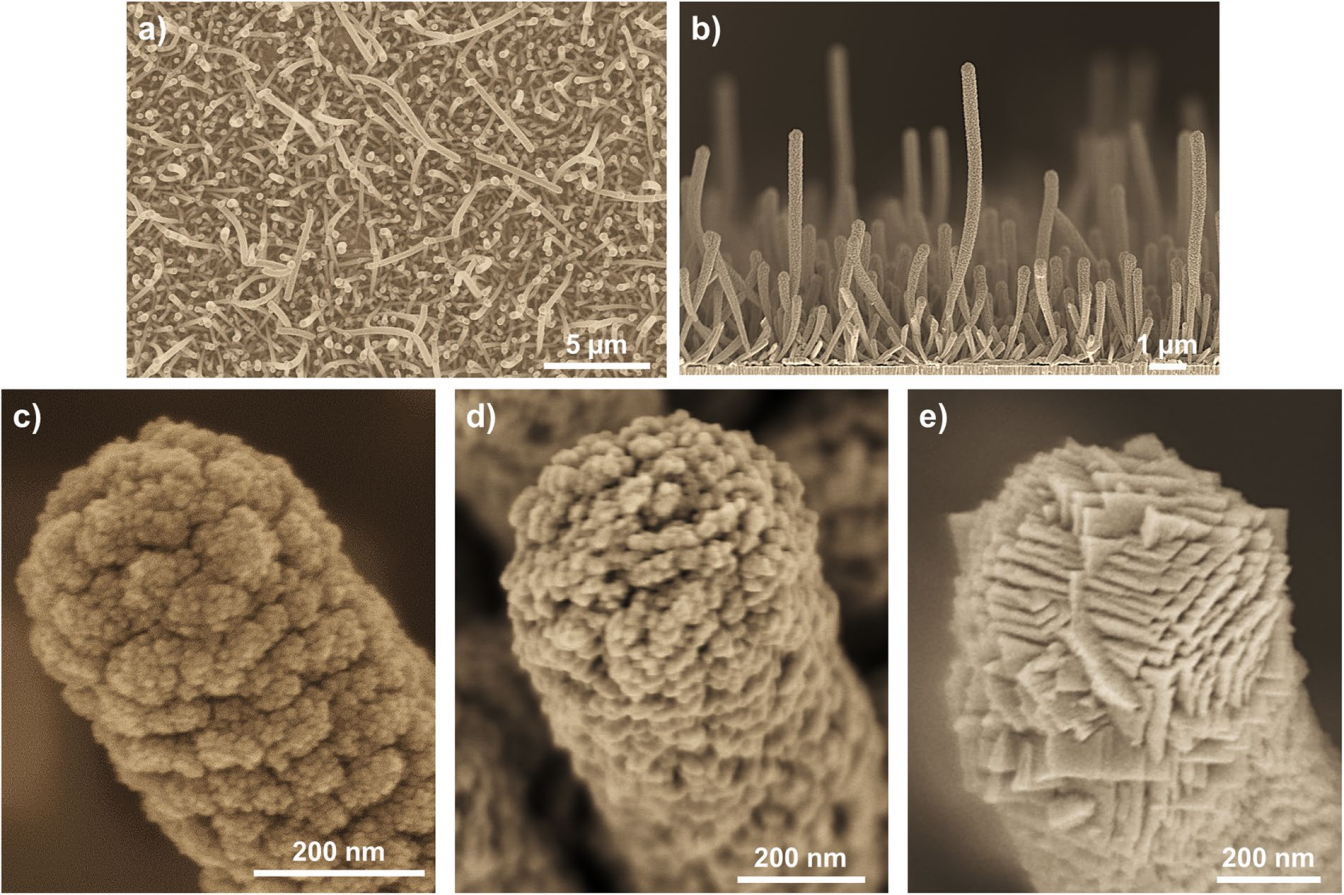
SEM normal view of ZnO@50 nm anatase NTs (**a**) and cross sections of ZnO@50 nm anatase NTs (**b**); high magnification micrographs showing the nanostructure of a ZnO@50 nm anatase NT (**c**), a 600 nm ZnO NT (**d**) and a 600 nm anatase NT (**e**).

Figure 1 shows the density (Fig. 1a), preferential vertical alignment (Fig. 1b) and microstructure (Fig. 1b) of 250 nm wall thickness ZnO NTs coated with a 50 nm equivalent thin film thickness of anatase (ZnO@50 nm anatase NTs). Note that equivalent thin film thickness refers to the same amount of material obtained in the absence of NWs as in Figure S1a–c. The porous nature of the equivalent thin film can be appreciated in Figure S1a–c in the Supplementary Information. For comparison a thicker ZnO NT also grown at room temperature (Fig. 1d) and an anatase NT grown at 250 °C (Fig. 1e), both of 600 nm equivalent thin film thickness, are shown. Even for higher thicknesses the ZnO exhibits a columnar microstructure (Fig. 1d) while anatase NTs present clear crystalline facets (Fig. 1e). The TiO_2_ coated ZnO NTs present a columnar microstructure regardless of the thickness of the TiO_2_ layer (in the 5–50 nm studied range) or the crystalline character of this outer layer. For the sake of simplicity, TiO_2_ deposited at room temperature will be referred as meso-TiO_2_, which is amorphous as shown by Borrás *et al*.^33^.

**Figure 2:**
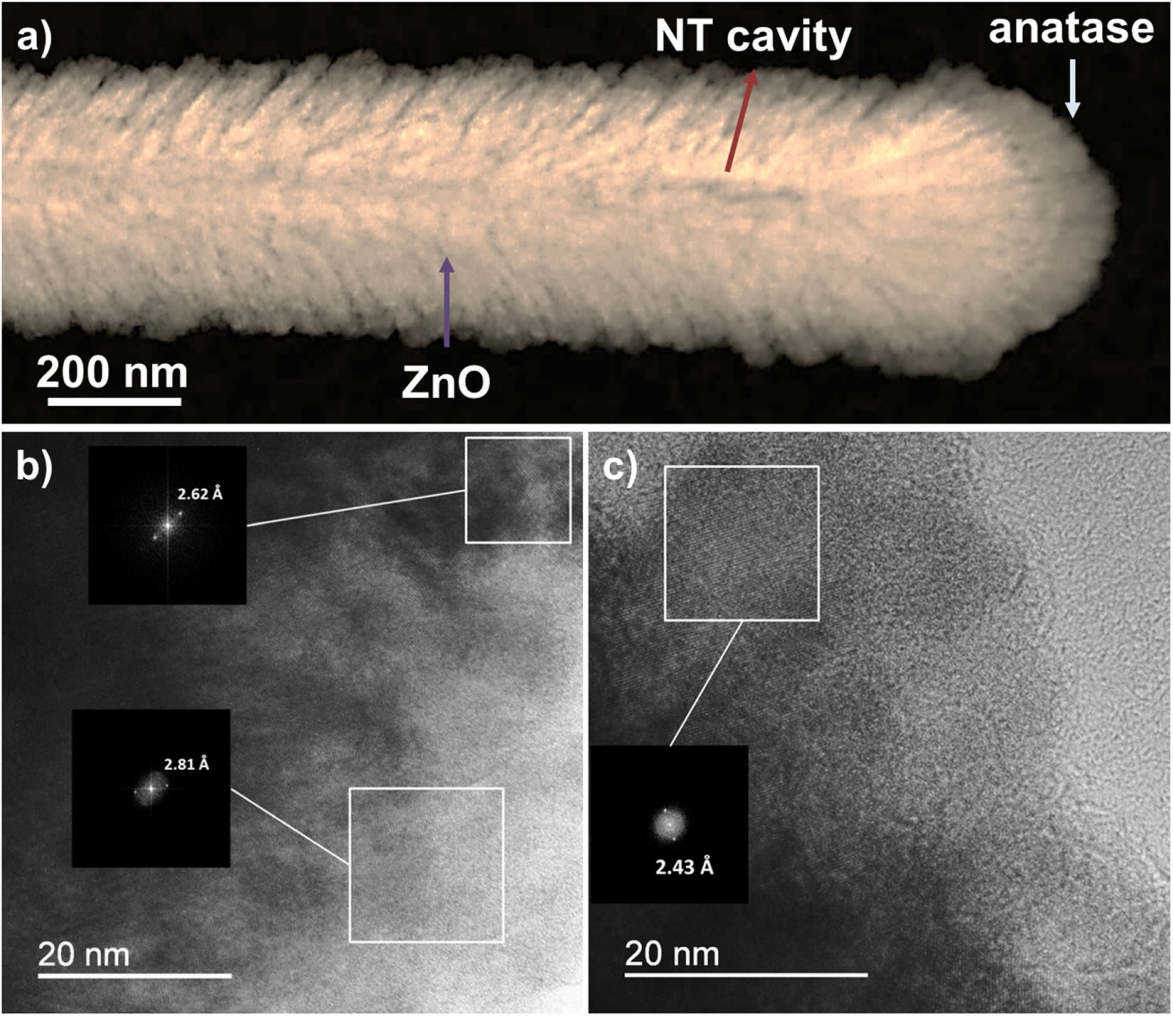
(**a**) HAADF-STEM image of a ZnO@50 nm anatase NT. (**b**,**c**) HRTEM images of 2 different areas of the NT and FFTs (insets) of some selected areas.

The stability and crystallinity of ZnO@TiO_2_ was studied by means of HRTEM, finding that the as-prepared samples stored under ambient conditions for roughly 2–3 months showed no indication of degradation of the inner ZnO as observed by Yang *et al*.^32^. As evidenced in Fig. 2a for a ZnO@50 nm anatase NT, the ZnO core is preserved and even its polycrystalline nature is still evident from the fast Fourier transform (FFT) of the selected areas in Fig. 2b, which gives plane distances of 2.81 Å and 2.62 Å, matching the ZnO wurtzite planes (100) and (002), respectively, in line with our previous results^1^. On the other hand, according to the calculated plane distance of 2.43 Å from FFT in Fig. 2c, anatase tends to crystallize preferentially in the (004) direction. This indicates that anatase TiO_2_ is formed exposing {001} facets which have been recently demonstrated to enhanced the photocatalytic activity and efficiency in dye-sensitized solar cells based on anatase nanoparticles^34, 35^.

**Figure 3:**
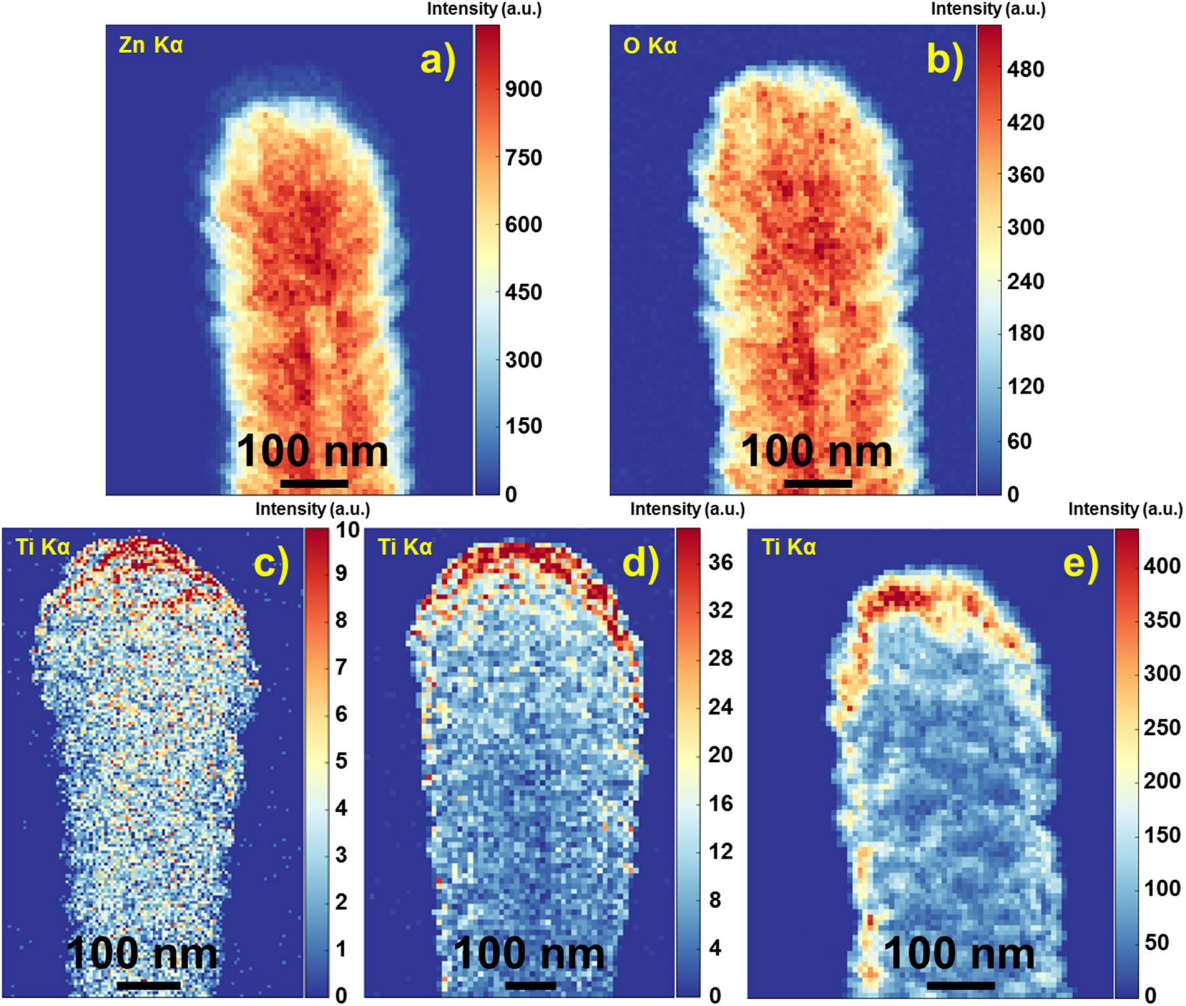
Zn Kα at 8.63 keV (**a**) and O Kα at 0.525 keV (**b**) in the EDX maps from the ZnO@50 nm meso-TiO_2_ NTc-e) Ti Kα at 4.51 keV in the resulting EDX maps obtained from the ZnO@meso-TiO_2_ NT for a TiO_2_ thickness of (**c**) 5 nm, (**d**) 20 nm and (**e**) 50 nm.

The evolution of the meso-TiO_2_ (grown at room temperature) or anatase (grown at 250 °C) coverage of a ZnO NT was investigated by STEM-EDX as shown in Fig. 3. The thickness of TiO_2_ shell was limited to 50 nm due to the final application pursued in this work. The integrity of the ZnO core is again demonstrated by the EDX map in Fig. 3a for a ZnO@50 nm-meso-TiO_2_ NT, whereas the map in Fig. 3b represents the total oxygen bonded to Zn and Ti. The EDX maps of titanium for ZnO@meso-TiO_2_ with TiO_2_ shells between 5–50 nm are presented in Fig. 3c–e. Note that the intensity scale of each EDX map in Fig. 3c–e is proportional to the amount of TiO_2_ present in the NTs (the measurements were performed under identical conditions), going from a maximum value of 10 for 5 nm (Fig. 3c) to more than 400 for 50 nm (Fig. 3e) of TiO_2_. Furthermore, these EDX maps were practically the same for anatase, data not shown here. With a layer only 5 nm thin (Fig. 3c), the intensity of Ti in the EDX maps is extremely low, revealing that the deposition occurred preferentially at the top due to self-shadowing effects as reported by Macias-Montero *et al*.^3^. Owing to this low TiO_2_ coverage, most of the ZnO is expected to be exposed to the environment. In the case of 20 nm (Fig. 3d), there are only a few regions in the NT with zero to very low intensity (dark blue in the color scale), meaning that almost no ZnO will be directly exposed. For 50 nm (Fig. 3e) a continuous layer of TiO_2_ is formed, hindering the direct contact between ZnO and the environment. It must not be forgotten that both meso-TiO_2_ and anatase are porous materials, so it is expected that both electrolyte and dye in a DSC diffuse to and into ZnO. However, due to this TiO_2_ coverage, it is also expected that this effect is diminished.

### Dye loading capabilities of ZnO-TiO_2_ systems

In order to investigate the influence of the multishell in the adsorption capacity, several adsorption-desorption experiments were conducted in the corresponding single layers TFs of ZnO, meso-TiO_2_ and anatase. From Figure S2 it is clear that the dye intake ability of ZnO was limited, perhaps due to the lower dye immersion time used to avoid damaging the oxide nanostructure. Meso-TiO_2_ performed much better, but it was greatly surpassed by anatase. Note that even though anatase and meso-TiO_2_ films were thicker, these conclusions will still be valid for thinner films since the absorption curves were normalized with respect to thickness. However changes in the porosity as a function of thickness cannot be completely ruled out.

**Table 1:** N719 dye concentration for the studied samples obtained from adsorption-desorption experiments.

Sample	Absorbance at 515 nm	Surface concentration[x10^−10^ moles/cm^2^]	Normalized surface concentration[x10^−12^ moles/nm.cm^2^]
ZnO 250 nm/5 nm meso-TiO_2_	0.0773	71.5 ± 1.4	28.05 ± 0.54
ZnO 250 nm/20 nm meso-TiO_2_	0.0896	82.9 ± 1.6	30.71 ± 0.60
ZnO 250 nm/50 nm meso-TiO_2_	0.1075	99.5 ± 1.9	33.16 ± 0.64
ZnO 250 nm/5 nm anatase	0.0983	91.0 ± 1.8	35.67 ± 0.69
ZnO 250 nm/20 nm anatase	0.1314	121.6 ± 2.4	45.03 ± 0.87
ZnO 250 nm/50 nm anatase	0.1662	153.8 ± 3.0	51.26 ± 0.99

In the case of multilayers of ZnO/TiO_2_ it was found that the addition of a TiO_2_ layer (both meso-TiO_2_ and anatase) to 250 nm ZnO significantly enhanced the dye loading capability of the films. Moreover, the effect resulted more pronounced in anatase than in amorphous meso-TiO_2_ as observed in the UV-Vis spectra from Figure S3. Table 1 gathers the obtained dye concentrations for the different multilayers (readers are referred to the SI section for the concentration determination procedure, see also Fig. S4 for the N719 concentration calibration curve). It is remarkable that the combination of ZnO and TiO_2_ (both meso-TiO_2_ and anatase) boosted the dye load in the films. Looking at the obtained concentrations in the table, three observations can be made. First, all ZnO/TiO_2_ (both meso-TiO_2_ and anatase) films were able to adsorb more dye than pure ZnO films. Secondly, anatase seemed to work better than meso-TiO_2_ in the ZnO/TiO_2_ system. Lastly, an increase in the thickness of the TiO_2_ films (both meso-TiO_2_ and anatase) gave rise to an increment in dye surface concentration (Table 1), which may be due to the particular evolution of the TiO_2_ microstructure with the thickness along with a higher surface area due to size increase of the NTs.

**Table 2:** Photovoltaic parameters for ZnO@TiO_2_ NT-based DSCs as a function of the TiO_2_ (meso or anatase) wall thicknesses. - Mean photovoltaic parameters values and estimated errors have been obtained from data of three devices with the same configuration. A ZnO NT cell without TiO_2_ has been included for comparison.

Cell	J_sc_[mA/cm^2^]	V_oc_[mV]	Fill Factor	Efficiency[%]
250 nm ZnO NT^3^	1.5 ± 0.2	460 ± 20	50 ± 1	0.3 ± 0.1
ZnO 250 nm@meso-TiO_2_ 5 nm NT	0.8 ± 0.1	540 ± 5	44 ± 1	0.2 ± 0.1
ZnO 250 nm@meso-TiO_2_ 20 nm NT	0.8 ± 0.1	542 ± 5	47 ± 1	0.2 ± 0.1
ZnO 250 nm@meso-TiO_2_ 50 nm NT	0.8 ± 0.1	529 ± 7	49 ± 1	0.2 ± 0.1
ZnO 250 nm/meso-TiO_2_ 50 nm TF	0.4 ± 0.1	573 ± 13	40 ± 8	0.1 ± 0.1
ZnO 250 nm@anatase 5 nm NT	0.9 ± 0.1	579 ± 13	38 ± 1	0.2 ± 0.1
ZnO 250 nm@anatase 20 nm NT	1.0 ± 0.1	588 ± 8	43 ± 5	0.2 ± 0.1
ZnO 250 nm@anatase 50 nm NT	1.0 ± 0.2	635 ± 15	32 ± 1	0.2 ± 0.1
ZnO 250 nm/anatase 50 nm TF	0.6 ± 0.2	615 ± 1	38 ± 2	0.1 ± 0.1

**Figure 4:**
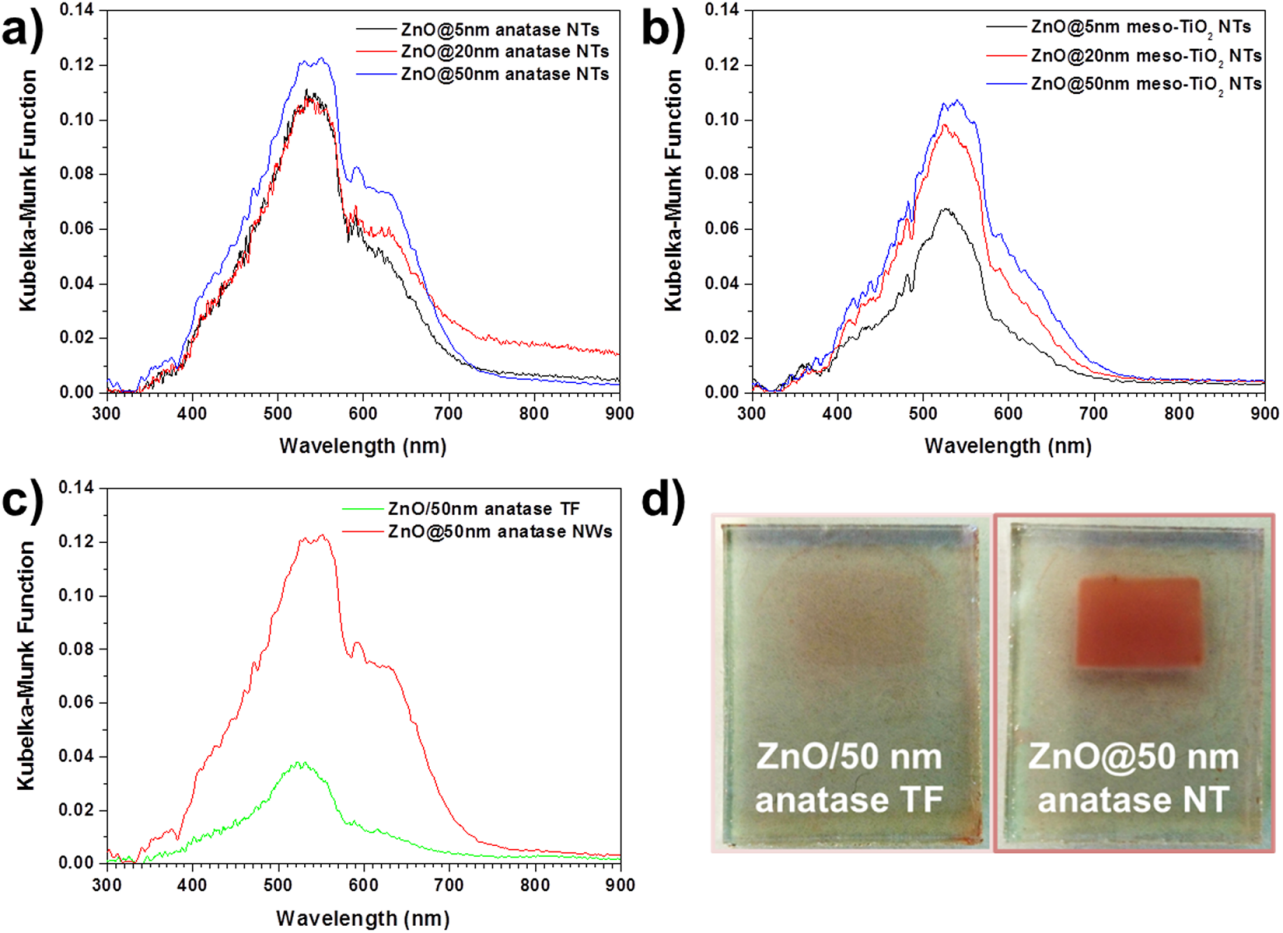
Kubelka-Munk function for (**a**) ZnO@anatase NTs, (**b**) ZnO@meso-TiO2 NTs and (**c**) comparison of the Kubelka-Munk function for a multishell NT (ZnO@50 nm anatase) cell and its multilayer analogue. (**d**) Electrodes for DSCs comprising ZnO/TiO_2_ TF (left) and ZnO@TiO_2_ NT (right) as active materials.

Due to severe limitations in the desorption of N719 from NTs (the films did not release all the dye), integrating sphere measurements were performed in ZnO samples covered with anatase (Fig. 4a), meso-TiO_2_ (Fig. 4b), and ZnO coated with 50 nm of anatase. Results are included in Fig. 4c to compare qualitatively the dye loading in thin film and nanotubes. The maximum observed in Fig. 4a–c, shifted from its position in solution (Fig. S3), is in good agreement with the determined absorption spectrum of N719 on TiO_2_^36^. It is clear from Fig. 4d, that NTs can increase substantially the dye loading by offering a much higher surface area as already observed for pure anatase systems. Moreover, this difference in dye concentration was readily noticeable at the naked eye as in Fig. 4b; the thin film samples possess a barely appreciable color whereas in NTs the dyed film is highly apparent.

### ZnO-TiO_2_ Dye sensitized solar cells

**Figure 5:**
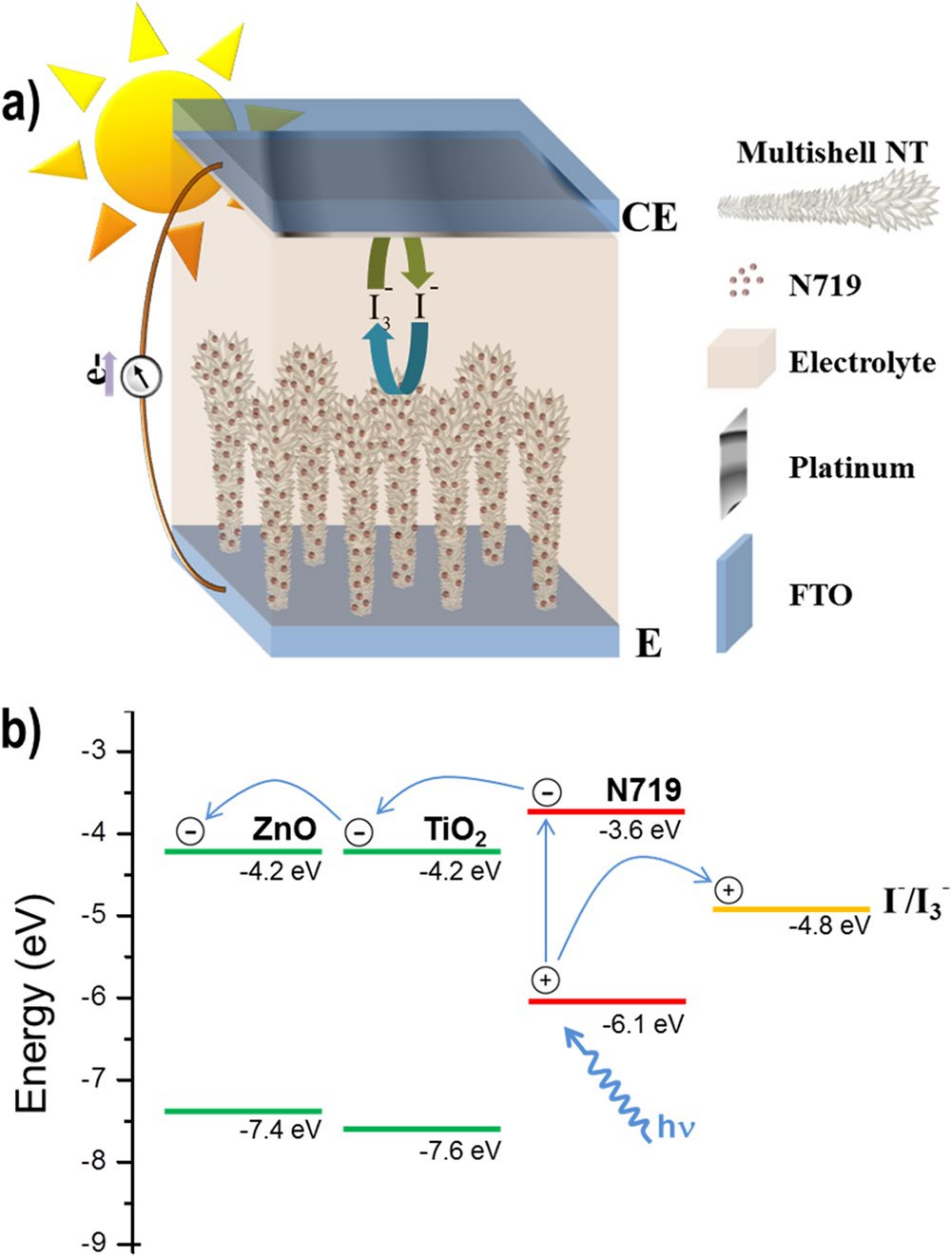
(**a**) Scheme of the solar cell and (**b**) Energy levels diagram for a ZnO/TiO_2_ system^43, 44^.

The main photovoltaic parameters of the fabricated DSCs depicted in Fig. 5a) are listed in Table 2. At first glance, the incorporation of a TiO_2_ shell was detrimental for the cell performance, giving rise to remarkably lower photocurrents compared to bare ZnO, in spite of the fact that the multishells showed a more intense dye adsorption capacity. However, the photovoltage became enhanced, which shows that the TiO_2_ shell does indeed improve the charge separation capability at the oxide/dye/electrolyte interface. This is a well-known fact related to the dielectric properties of both oxides^14, 37^. That the photoconversion process is now determined by the TiO_2_/dye/electrolyte system rather than by the ZnO/dye/electrolyte one, explains why the photovoltage, which is a direct measure of charge separation, is greater and basically the same regardless the thickness of the cell. Despite these promising features, the photocurrent worsened when the thickness was increased. This indicates that amorphous TiO_2_ is not a good electron conducting material, which also explains the lower fill factor due to high series resistance. As a matter of fact, when the shell is made of the anatase material, which is a more efficient electron conductor, not only the photovoltage but also the photocurrent is larger. Thin films with 50 nm of meso-TiO_2_ and anatase have been measured as references. These devices produced a much lower photocurrent due to lower light harvesting, as shown in Fig. 4c.

Furthermore, we can trace the origin of the low performance of the ZnO-TiO_2_ system by calculating the theoretical *J*_*SC*_ of the NT electrodes with the adsorbed dye via Equation 1

1 $${J}_{sc}=q{\int }_{{\lambda }_{min}}^{{\lambda }_{max}}{I}_{0}(\lambda )IPCE(\lambda )d\lambda $$

where *λ*_*min*_ and *λ*_*max*_ define the wavelength range and *I*_0_ is the AM1.5 solar flux. The IPCE can be expressed as the product of the efficiency of the processes involved in the electrical conversion in DSC as in Equation 2

2 $$IPCE(\lambda )={\eta }_{lh}\,(\lambda ){\eta }_{inj}\,(\lambda ){\eta }_{col}\,(\lambda )$$

where *η*_*lh*_(*λ*) is the light-harvesting efficiency of the sensitized oxide layer *η*_*inj*_(*λ*) is the electron injection efficiency from the sensitizer to the oxide, and *η*_*col*_(*λ*) is the electron collection efficiency (dye regeneration efficiency is implicitly included in the injection term). Assuming 100% injection and collection efficiency it is possible to calculate the theoretical *J*_*SC*_ from the light absorption data in Fig. 4. In this calculation it is also implicitly assumed that there are no losses due to reflection and absorption of the electrolyte. However, they are expected to be small and affect all samples in the same extent. Results are shown in Table ST1.

It is observed that the theoretical current is almost one order of magnitude larger that the experimental one. This indicates that the main loss in current is due to low injection and strong recombination. We also observe that anatase samples are more efficient in harvesting light, which explains the relatively larger photocurrents. In any case the results demonstrate that higher currents are potentially extractable from this kind of samples.

According to the energy diagram in Fig. 5, the conduction bands of ZnO and TiO_2_, and thus their respective Fermi levels, are generally considered to be aligned. However, the results indicate that the electronic transfer between TiO_2_ and ZnO is hindered due to the existence of a small energy barrier^26^. Owing to this lack of electronic transfer between TiO_2_ and ZnO, the photovoltaic response seems to be originated primarily from the ZnO cores, although a beneficial effect of the TiO_2_ core is observed in the photovoltage, possibly due to a blocking of recombination as explained above.

Comparing with results from the bibliography where core@shell nanostructures of the type ZnO@TiO_2_ have been synthetized and used in DSCs^27, 30, 31, 38, 39^, here the relatively thick ZnO blocking layer, which also acts as seed for the growth of the organic NWs (see methods), hinders any kind of direct contact between TiO_2_ and the photoanode, i.e. the photogenerated electrons in the TiO_2_ must travel through the ZnO first, and as mentioned before, if charge transfer from TiO_2_ to ZnO is hindered, then it is reasonable no to expect a photovoltaic improvement as observed. In the previous mentioned references^27, 30, 31, 38, 39^, both ZnO and TiO_2_ were at least partially in contact with the photoanode, observing an improvement of the performance of the cells. Nonetheless, it has been reported that only in the case of ultrathin ZnO blocking layers ( < 20 nm) there is an improvement in the overall efficiency of the cells, observing a deterioration of the photovoltaic performance with only 20 nm thick ZnO blocking layers^29^. Despite ZnO was grown by atomic layer deposition, even with this technique ZnO may present an island-like or columnar growth depending on the substrate and deposition conditions^40, 41^. This further supports our argument that for highly porous or quite thin layers of ZnO or TiO_2_, where both oxides are in contact with the photoanode, a general enhancement of the cells is most likely to occur due to the contribution of both oxides, while if only one of the oxides is contacting the photoanode, then a degradation of the efficiency is usually noticed. These general observations explain the discrepancies commonly observed in the literature.

## Conclusion

Vertically aligned multishell ZnO@TiO_2_ NTs with a high degree of conformality of each layer have been fabricated by a whole vacuum procedure. The exposed methodology allowed us to tune the chemical composition, crystallinity and thickness of the NTs which were implemented as photoanodes in DSCs. Moreover, the ability of the supported NTs to withstand the cells fabrication process demonstrated their robustness.

Mixed results were obtained for amorphous and crystalline TiO_2_, however, it has been found that the addition of a thin TiO_2_ shell turned out to be detrimental for the performance of the cells, decreasing the maximum attainable photocurrent but increasing the Open-circuit Voltage (V_OC_) for both amorphous and anatase shells. However, thicker anatase shells improve the value of V_OC_ up to a value of 635 mV for a 50 nm shell, whereas this parameter decreased monotonically with thicker meso-TiO_2_ NT walls up to a value of 460 mV (higher than that of bare 250 nm ZnO NTs). This behavior was explained in terms of the apparent negligible electronic transference between ZnO and TiO_2_, which taking into account the nature of the blocking layer and growth of both semiconductor oxides also explains the frequent discrepancies found in literature.

The fabrication of ZnO@TiO_2_ NTs presented here can be easily extended to other metal oxides by selecting the organometallic/metal-organic precursors. Furthermore, we have demonstrated that with the deposition methodology developed in this work, ZnO@TiO_2_ nanotubes with stable interfaces can be produced, paving the way for a broader range of applications such as photocatalysis, UV absorbers and antibacterial surfaces.

## Methods

The fabrication of the nanostructured photoanodes comprised a full-vacuum multi-step procedure which can be conveniently divided into three main steps as described in refs 1, 3.

### Growth of ONWs by OPVD

ONWs were grown on a thin film of ZnO or TiO_2_ (amorphous or anatase) previously deposited by PECVD on FTO coated glass substrates. Polished n-type Si(100) purchased from Topsil and fused silica from Sico Technology GmbH were used in each preparation for later characterization. The organic Phthalocyanine (H_2_Pc) was supplied from Aldrich and used as received without further purification. The OPVD procedure for the formation of single crystal ONWs has been fully described in refs 1–3, 42. It consists on the sublimation of the organic molecules from a Knudsen cell at 0.02 mbar of Ar using a growth rate about 0.3 Å/s and controlled substrate temperature. The substrates temperature was settled at ~230 °C for H_2_Pc. The nominal thickness of the NWs was set to 0.65 kÅ which corresponds to NWs 2–3 μm long.

### Growth of ZnO and TiO_2_ layers by PECVD

Both semiconducting oxides, ZnO and TiO_2_, were fabricated by PECVD in a microwave (2.45 GHz) ECR reactor with a down-stream configuration as described in ref. 1. Diethylzinc (ZnEt_2_) and titanium tetraisopropoxide (TTIP) were utilized as precursors (Sigma Aldrich). Crystalline ZnO was grown at RT with oxygen as plasma gas. Total pressure in the chamber was settled at 1.5 × 10^−2^ mbar and plasma power at 400 W. meso-TiO_2_ was grown at the same conditions with a slightly lower pressure (8.6 × 10^−3^ mbar). Anatase thin films and nanotubes were prepared as meso-TiO_2_ but heating the substrates at 250 °C during the fabrication process. In both cases the thickness of the ZnO was fixed to 250 nm and three different TiO_2_ thicknesses were employed: 5 nm, 20 nm and 50 nm. Thin films of TiO_2_ (amorphous or anatase)/ZnO grown under identical conditions to the nanotubes but avoiding the ONW template have been used as references and will be referred here as TF(s).

### Empty of the 1D nanostructures

Under standard conditions a heating treatment at 350 °C and 10^−6^ mbar of pressure was applied to these samples for 3 hours to achieve a complete emptying of the inner organic core. No alteration of the vacuum was detected during the process. After the annealing process is performed, the samples were allowed to cool down in high vacuum avoiding water condensation in the highly porous nanotube walls.

### Solar cells fabrication procedure

Counter electrodes. FTO/glass substrates of 2.5 × 2 cm^2^ provided by Xop Glass (12–14 Ω/γ) were drilled in two points for later electrolyte injection, rinsed with acetone, isopropanol and absolute ethanol and heated to 500 °C for 1 hour. 12 μL of plastisol (Solaronix) are dispersed on the substrates, dried in air and heated in a furnace for 20′ at 400 °C.

Working electrodes. FTO/glass substrates were cleaned just as the counter electrodes. An active area of 0.7 cm^2^ was defined with an aluminum foil mask and a layer of less than 100 nm of ZnO was deposited by PECVD. This ZnO film acts as a hole blocking layer and provides the necessary roughness for the growth of ONWs. ZnO NTs with different thicknesses were fabricated by PECVD onto the FTO electrode through a mask to delimitate a covered area of 7 × 10 mm^2^. Samples were heated up to 120 °C before immersing in the dye solution, 0.5 mM solution of N719 dye (cis-diisothiocyanato-bis(2,20-bipyridyl-4,40-dicarboxylato) ruthenium(II) bis (tetrabutylammonium)) [purchased from Solaronix] in ethanol, to prevent adsorption of air moisture. The immersion time was limited to 1 hour in all cases.

Electrolytic solution. It was prepared by addition of 0.6 M 1,2-dimethyl-3-propylimidazole iodine (DMPII), 0.1 M LiI, 0.5 M 4-tertbutyl-pyridine (TBP), 0.05 M I_2_ and 0.1 M guanidinium thiocyanate (GuSCN) to a mixture of acetonitrile/valeronitrile (85/15).

Sealing of the cells. A frame of a thermoplastic polymer (Surlyn, Solaronix) was placed on the perimeter of the active area and then sandwiched with the counterelectrode. The whole cell was heated to 140 °C under slight pressure to ensure a proper sealing. After that the electrolyte was injected and the holes on the counterelectrode sealed with Surlyn and a cover slide glass.

### Dye N719 concentration determination

A calibration curve was constructed by measuring the absorbance between 200 and 900 nm of four solutions of dye N719 in KOH 1 M in MeOH, being the molar concentration of the dye in each case 1 × 10^−6^, 5 × 10^−6^, 5 × 10^−5^ and 1 × 10^−4^. Using the Lambert-Beer’s law, the absorption coefficient ε (M^−1^cm^−1^) can be calculated by taking the absorbance at 515 nm. In this case, a straight line with R2 = 0.997 was obtained by linear regression, estimating a value of 11331 ± 276 for ε. With this value and a known value of 1 cm for the light path, the molar volume concentration was calculated for each sample. The total number of moles for each film was calculated by multiplying the obtained concentration by the volume of solution employed (2 ml). Then, the surface concentration was calculated by dividing this value by the area of the sample (1.875 cm^2^). Finally, the normalized surface concentration is simply this value divided by the thickness of the layer.

A precision quartz cell from Hellma with a light path of 1 cm and a Cary 100 spectrometer from Varian were used for these experiments.

The UV-Visible spectra of dye-sesitized electrodes were measured using an integrating sphere (Mikropack, ISP-50–8-R-GT) in the range 350–700 nm

### Experimental characterization methods

The solar-cell devices were characterized using a solar simulator with an AM1.5 G filter (ABET). A reference solar cell with temperature output (Oriel, 91150) was used for calibration.

SEM micrographs were acquired in a Hitachi S4800 working at 2 kV. The samples were dispersed onto Holey carbon films on Cu or Ni grids from Agar scientific for TEM characterization. EDX maps were acquired with a FEI Tecnai Osiris TEM/STEM 80–200 working at 200 kV. Post-processing of EDX data was performed with the open source Hyperspy software: hyperspy.org. as described elsewhere^7^. HAADF-STEM and HRTEM were carried out with both Osiris and FEI Tecnai G2F30 S-Twin STEM microscope also working at 200 kV.

## Electronic supplementary material

supplementary info
