# Supplementary material for: One-reactor plasma assisted fabrication of ZnO@TiO2 multishell nanotubes: assessing the impact of a full coverage on the photovoltaic performance
Source: Sci Rep. 2017 Aug 29;7:9621. doi: 10.1038/s41598-017-09601-7 (PMC5575108; doi:10.1038/s41598-017-09601-7)
Supplement: Supplementary file 1 — supplementary info [file 41598_2017_9601_MOESM1_ESM.doc]

**Supplementary Information**

**One-reactor plasma assisted fabrication of ZnO@TiO_2_ multishell nanotubes: assessing the impact of a full coverage on the photovoltaic performance**

Alejandro Nicolas Filippin, Manuel Macias-Montero, Zineb Saghi, Jesús Idígoras, Pierre Burdet, Juan R. Sanchez-Valencia, Angel Barranco, Paul Migdley, Juan A. Anta & Ana Borras


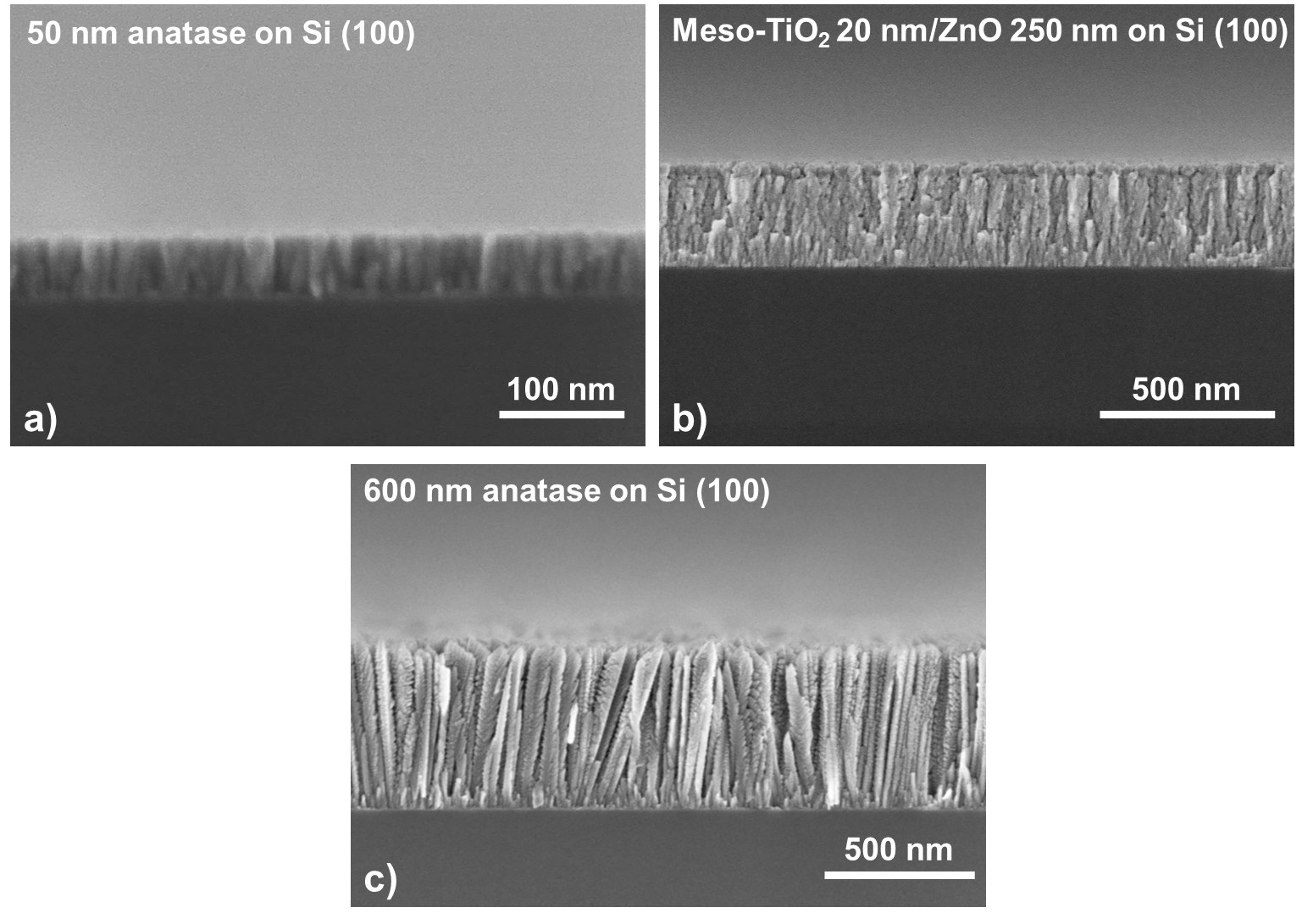


**Figure S1**. SEM cross section of a) 50 nm thick anatase layer, b) meso-TiO2 20 nm/ZnO 250 nm and c) anatase 600 nm.

In the case of the single layer adsorption-desorption experiments, the samples were immersed in dye N719 followed by desorption of the dye in KOH 1M in MeOH and collection of the UV-Vis spectra of the resulting dye solutions. Figure S2 shows the UV-Vis spectra in the region of one of the absorption bands of N719 for ZnO, meso-TiO_2_ and anatase. Note that the absorbance has been normalized by the corresponding thin film thickness.


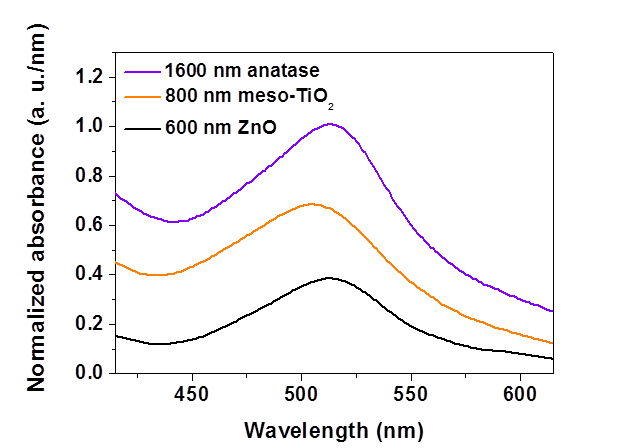


**Figure S2**. UV-Vis spectra of N719 in KOH 1M in MeOH after desorption from ZnO, meso-TiO_2_ and anatase thin films. The absorbance has been normalized to the thickness of each sample.

UV-Vis absorption spectra for N719 adsorbed onto ZnO/TiO_2_ thin films are presented in Figure S3. Note that the normalized surface concentration is just the surface concentration divided by the thickness of the film


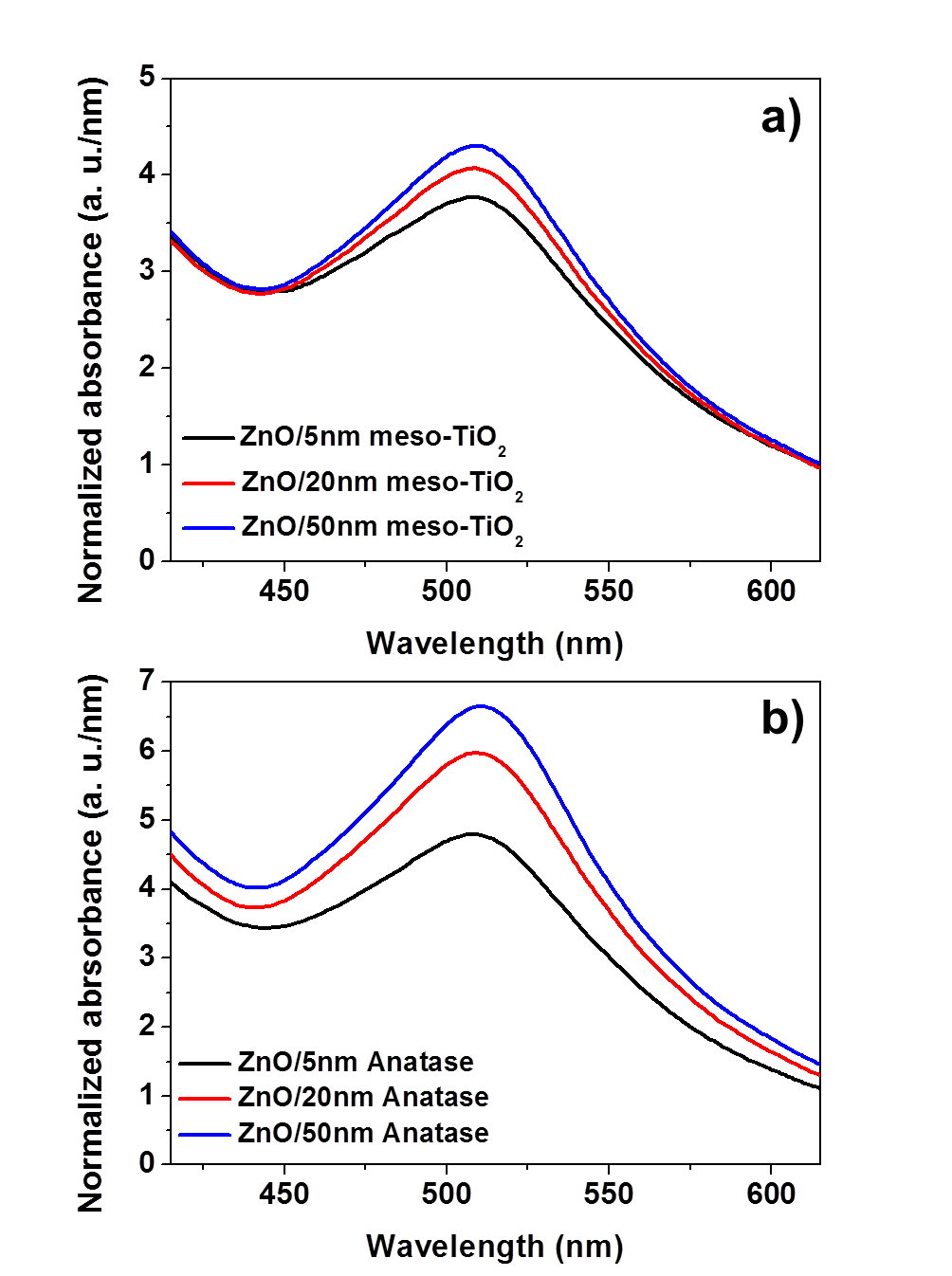


**Figure S3**. UV-Vis spectra of N719 in KOH 1M in MeOH after desorption from (a) ZnO/meso-TiO_2_ and (b) ZnO/anatase.

A calibration curve was constructed by measuring the absorbance between 200 and 900 nm of four solutions of dye N719 in KOH 1M in MeOH, being the molar concentration of the dye in each case 1x10^-6^, 5x10^-6^, 5x10^-5^ and 1x10^-4^. Using the Lambert-Beer´s law, the absorption coefficient
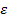
 (M^-1^cm^-1^) was calculated by taking the absorbance at 515 nm. This law is usually expressed as
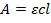
, where *A* is the absorbance,
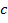
 the concentration (M) and
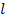
 the light path (cm). In this case, a straight line with R^2^ = 0.999 (Fig. S4) was obtained by linear regression, estimating a value of 11528±224 for
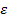
. With this value and a known value of 1 cm for the light path, the molar volume concentration was calculated for each sample.


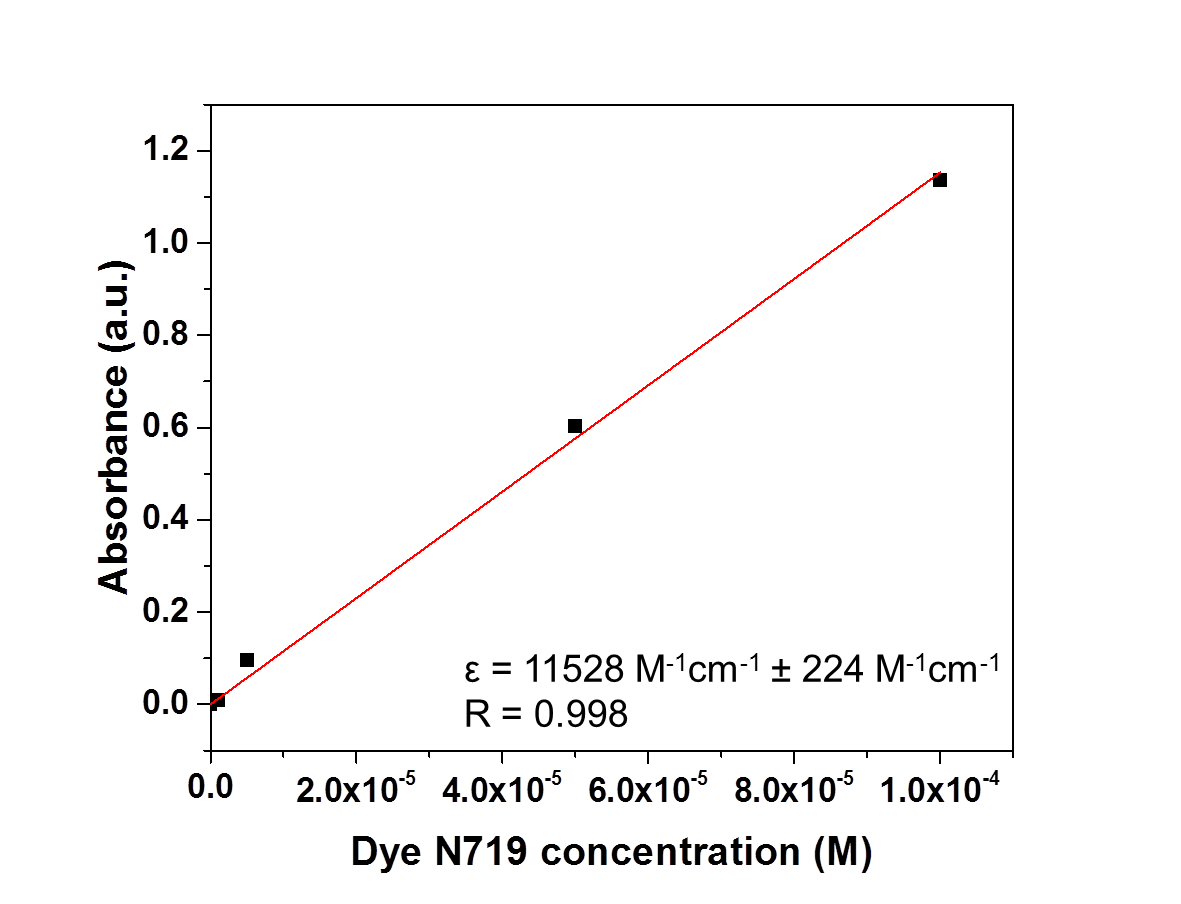


**Figure S4**. Calibration curve for N719 in KOH 1M in MeOH.

The total number of moles for each film was calculated by multiplying the obtained concentration by the volume of solution employed (2 ml). Then, the surface concentration was calculated by dividing this value by the area of the sample (1.875 cm^2^). Finally, the normalized surface concentration is simply this value divided by the thickness of the layer.

A precision quartz cell from Hellma with a light path of 1 cm and a Cary 100 spectrometer from Varian were used for these experiments.


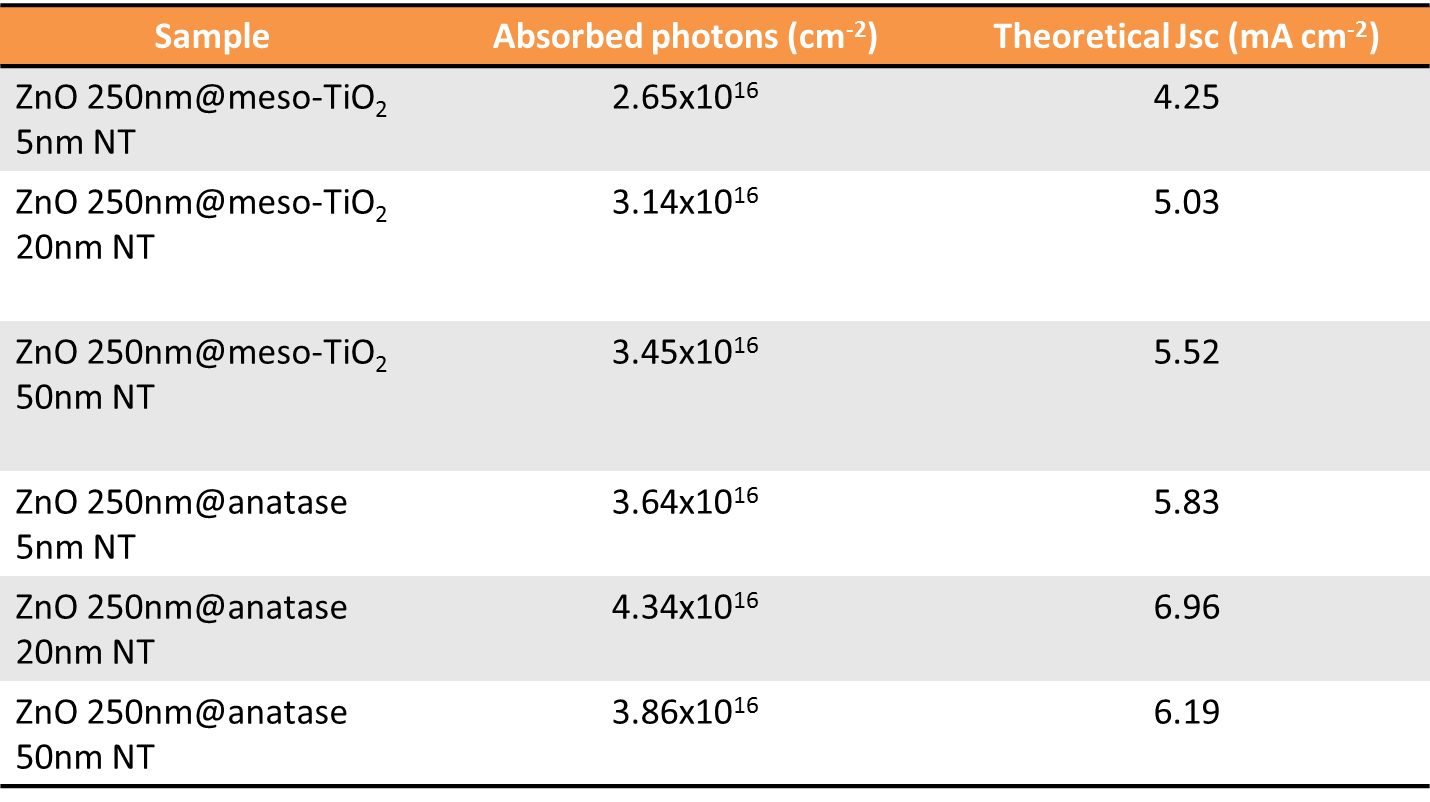


Table ST1. Absorbed photons for meso-TiO2 and anatase nanotubes samples with adsorbed N719, calculated from the reflectivity spectra in Figure 4a-b and the photon flux of AM1.5 assuming zero transmittance, and theoretical Jsc considering 100% collecting efficiency and injection efficiency.
